# Impact of Extent of Resection on Survival in Brain Metastasis: An Analysis of 867 Patients

**DOI:** 10.1227/neu.0000000000003544

**Published:** 2025-06-23

**Authors:** Alexander F. C. Hulsbergen, Francesca Siddi, Christian Cerecedo, Faith C. Robertson, Yu-Tung Lo, Vasileios Kavouridis, Felix Ehret, Charissa A. C. Jessurun, Ishaan R. Tewarie, John G. Phillips, Joost J. C. Verhoeff, Timothy R. Smith, Marike Broekman

**Affiliations:** * Computational Neuroscience Outcomes Center, Department of Neurosurgery, Brigham and Women's Hospital, Harvard Medical School, Boston, Massachusetts, USA;; ‡ Departments of Neurosurgery, Haaglanden Medical Center and Leiden University Medical Center, Leiden University, Leiden, Zuid-Holland, The Netherlands;; § Department of Neurosurgery, University Medical Center Utrecht, Utrecht, The Netherlands;; ‖ Section of Neurosurgery, Department of Neurosciences, Biomedicine and Movement Sciences, University of Verona, Verona, Italy;; ¶ Department of Neurosurgery, Massachusetts General Hospital, Boston, Massachusetts, USA;; # Department of Neurosurgery, National Neuroscience Institute, Singapore, Singapore;; ** Department of Neurosurgery, St. Olavs Hospital, Trondheim, Norway;; †† Department of Radiation Oncology, Massachusetts General Hospital, Harvard Medical School, Boston, Massachusetts, USA;; ‡‡ Department of Radiation Oncology, Charité – Universitätsmedizin Berlin, Corporate Member of Freie Universität Berlin and Humboldt-Universität zu Berlin, Berlin, Germany;; §§ German Cancer Consortium (DKTK), partner site Berlin, a partnership between DKFZ and Charité – Universitätsmedizin, Berlin, Germany;; ‖‖ Division of Radiation Oncology, Tennessee Oncology, Nashville, Tennessee, USA;; ¶¶ Department of Radiation Oncology, University Medical Center Utrecht, Utrecht, The Netherlands;; ## Department of Neurology, Massachusetts General Hospital, Boston, Massachusetts, USA

**Keywords:** Brain metastasis, Extent of resection, Intracranial progression-free survival, Magnetic resonance imaging, Neurosurgery, Overall survival

## Abstract

**BACKGROUND AND OBJECTIVES:**

The association between extent of resection (EOR) and outcomes after brain metastasis (BM) surgery remains unclear. The aim of this study was to examine the relation between EOR and overall survival (OS)/intracranial progression-free survival (IC-PFS) in this patient group.

**METHODS:**

We included patients who underwent BM resection and had postoperative MRI <72 hours. Presence of any residual on MRI was defined as subtotal resection (STR) as opposed to gross total resection. Multivariable analyses were adjusted for known confounders. Post hoc analysis explored the effect size across different subgroups. A secondary outcome was the occurrence of leptomeningeal disease (LMD).

**RESULTS:**

We included 867 patients; median age was 61 years (IQR 53-68), and median BM diameter was 3.0 cm (IQR 2.2-3.9). Extracranial metastases were present in 310 (35.8%) patients, and 365 (42.1%) received stereotactic radiosurgery (SRS) to the resection cavity. No residual on MRI was present in 345 patients (39.9%). In multivariable analysis, STR correlated with decreased IC-PFS (hazard ratio [HR] 1.32, 95% CI 1.13-1.55, *P* < .001) and OS (HR 1.28, 95% CI 1.08-1.53, *P* = .005) and a higher occurrence of LMD (odds ratio 1.74, 95% CI 1.10-2.76, *P* = .02). STR + SRS also correlated with decreased IC-PFS compared with gross total resection (HR 1.35, 95% CI 1.02-1.66, *P* = .04). In subgroup analysis, the strongest association between EOR and outcomes was observed in large (>3 cm) BMs, supratentorial BMs, and patients without extracranial disease. The use of cavity SRS or the number of BMs had little impact on this association.

**CONCLUSION:**

Residual tumor on postoperative MRI after BM resection correlated with worse IC-PFS and OS after adjusting for confounding variables. An increased prevalence of LMD may be a possible mechanism through which these patients experience worse outcomes.

**ABBREVIATIONS:**
BM: Brain metastasis
EOR: Extent of resection
GTR: Gross total resection
HR: Hazard ratio
IC-PFS: Intracranial progression-free survival
LMD: Leptomeningeal disease
OS: Overall survival
SRS: Stereotactic radiosurgery
STR: Subtotal resection
WBRT: Whole-brain radiotherapy.

Neurosurgical resection has been a cornerstone in the treatment of brain metastasis (BM) for several decades.^1,2^ Typically, a surgeon aims to remove all tumor tissue from the surrounding parenchyma, thereby achieving gross total resection (GTR). Despite modern advances in neuronavigation, residual tumor may still be encountered on postoperative MRI, constituting subtotal resection (STR). Although the impact of the extent of resection (EOR) has been thoroughly investigated for other tumors such as low^3^ or high-grade^4^ glioma, evidence of its importance for BM is less clear. The Congress of Neurological Surgeons 2019 guidelines on BM treatment give a level III recommendation of GTR over STR in recursive partitioning class I patients (<65 years, no extracranial metastases, primary tumor under control, and Karnofsky Performance Status >70), but refrains from recommendations for other patient.^5^ The heterogeneous nature of BMs regarding number, size, primary tumor origin, extracranial disease, and other factors presents challenges for generalizing these recommendations.

Therefore, we sought to investigate the association between the EOR and overall survival (OS)/intracranial progression-free survival (IC-PFS) in patients with BM who underwent neurosurgical resection.

## METHODS

Patients were identified through an institutional BM database of the Brigham and Women's Hospital, Department of Neurosurgery, after obtaining institutional review board approval (Partner's Institutional Review Board #2015P002352). Because this was a retrospective review, patient consent was not required by our review board. Patients were included if they underwent resection of BMs between January 2004 and January 2018 and had an MRI performed within 72 hours postoperatively.

Any residual contrast enhancement on postoperative MRI was recorded. Residuals were classified by shape (linear, nodular, or irregular). Residuals were determined by measuring T1 postcontrast enhancement adjacent to the surgical cavity and subtracting precontrast enhancement size. This was performed by 4 authors (FS, FCR, CC, AFCH) after receiving training from a neurosurgery fellow (FS) and an attending neurosurgeon (MB). In case of uncertainty, the neurosurgery fellow (FS) was consulted to make a final decision. The presence of any residual on postoperative MRI, regardless of shape or size, was classified as STR; the remaining cases were classified as GTR. If a patient had multiple brain metastases, EOR was used to describe the resected lesion exclusively.

Primary outcomes of interest were OS (time between craniotomy and death or loss to follow-up) and IC-PFS (time between craniotomy and radiological intracranial progression, death, or loss to follow-up). The impact of EOR on these outcomes was measured in multivariable analysis after adjustment for patient age, performance status, number of BMs, size of the resected BM, infratentorial vs supratentorial location, primary tumor origin, extracranial metastases, presence of a targetable mutation, newly diagnosed vs recurrent BM status, year of surgery, and adjuvant radiation.

Secondary analyses included the impact on OS/IC-PFS of different residual shapes on OS and IC-PFS, intraoperative surgeon-assessed EOR, the association between EOR and the occurrence of leptomeningeal disease (LMD), and the effect size of our primary analysis in different clinical subgroups.

Statistics were performed in R version 4.1.2 (R Foundation for Statistical Computing). Counts, percentages, medians, and IQR were used to describe the properties of the data. Survival analyses were performed using Cox Proportional Hazards models and Kaplan-Meier curves; significance (*P* < .05) in these was determined using the Log-Rank test. The association between EOR and LMD was assessed using logistic regression.

## RESULTS

### Baseline Characteristics

Out of 1070 patients in our database, 914 (85.4%) had MRI <72 hours after BM resection; 47 (4.4%) were excluded because of lack of MRI interpretability (eg, no contrast administration, motion artifact, or hemorrhage in the resection cavity). Thus, 867 patients were included.

Baseline characteristics are presented in Table 1. Median age was 61 years (IQR 53-68); median diameter of the resected BM was 3.0 cm (IQR 2.2-3.9). The number of BMs per patient was 1 (n = 431, 49.7%), 2 (n = 173, 20.0%), 3 (n = 87, 10.0%), or more than 3 (n = 176, 20.3%). Localization was supratentorial in 669 patients (77.1%). Extracranial metastases were present in 310 (35.8%). Adjuvant radiation comprised stereotactic radiosurgery (SRS) (n = 365, 42.1%), whole-brain radiotherapy (WBRT) (n = 343, 39.6%), or none (n = 159, 18.3%). Postoperative SRS gradually replaced WBRT over the course of our study period, especially in patients with fewer/smaller BMs. **Supplemental Digital Content 1** (http://links.lww.com/NEU/E821) outlines the differences between these groups and the reasons for deferring radiation. Recurrent metastases were resected in 103 (11.9%) patients. The timing of MRI after surgery was <48 hours in 849 (98.0%) patients.

**TABLE 1.** Baseline Characteristics

Variable	Overall (N = 867)
Male	360 (41.5)
Age (median, IQR)	61 (53-68)
BM size (median, IQR)	3.0 (2.2-3.9)
Resected BM location	
Supratentorial	669 (77.1)
Infratentorial	198 (22.9)
No. of brain metastases	
1	431 (49.7)
2	173 (20.0)
3	87 (10.0)
>3	176 (20.3)
Extracranial metastases	310 (35.8)
Primary tumor origin	
Breast	123 (14.2)
Colorectal	28 (3.2)
Gynecological	51 (5.9)
Lung	388 (44.8)
Melanoma	116 (13.4)
Miscellaneous	122 (14.1)
Renal	39 (4.5)
Karnofsky performance status (median, IQR)	80 (80-100)
Recurrent BM	103 (11.9)
Adjuvant radiation	
SRS	365 (42.1)
WBRT	343 (39.6)
None	159 (18.3)
Year of diagnosis	
2004-2009	195 (22.5)
2010-2014	390 (45.0)
2015-2018	282 (32.5)

BM, brain metastasis; SRS, stereotactic radiosurgery.

Median OS was 14.5 (IQR 5.8-36.1) months, and median IC-PFS was 6.6 (IQR 3.5-14.6) months, with 395 (45.6%) patients experiencing intracranial recurrence. In patients who were not deceased at the last follow-up (n = 297, 34.3%), the median follow-up was 15.6 (IQR 7.1-34.8) months.

### Postoperative Residual

On postoperative MRI, 345 (39.9%) patients had no residual whatsoever, while the remainder had linear (n = 259, 29.9%), nodular (n = 241, 27.9), or irregularly shaped (n = 20, 2.3%) residual. In the linear group, 73 patients had minimal linear enhancement adjacent to the resection cavity that was deemed dubious for residual disease by the reporting radiologist. To make a conservative estimation, these were classified as residuals in the main analysis.

Based on operative notes, all visible tumor was removed in 787 (90.8%) patients. In 73 (8.4%) patients, residual was described that was not removed. Six (0.7%) operative notes were inconclusive, and one was unavailable.

Table 2 presents the overlap between MRI-determined and surgeon-assessed EOR. Of 787 patients with intraoperatively assessed GTR, 458 (58.3%) had residual on MRI, which was nodular in 198 (25.2%) cases. In 73 patients with intraoperatively assessed STR, 57 (78.0%) had residual on MRI, which was nodular in 39 (53.4%) cases.

**TABLE 2.** Definitions of EOR

MRI residual	N (%)	Intraoperative GTR (%)	Intraoperative STR (%)	Operative report inconclusive (%)
All cases	867	787	73	6
Shape of residual				
Absence of any residual (GTR by study definition)	345 (39.9)	327 (41.7)	16 (21.9)	1 (16.7)
Irregular/ambiguous	20 (2.3)	19 (2.4)	1 (1.4)	0 (0.0)
Linear	259 (29.9)	241 (30.7)	17 (23.3)	1 (16.7)
Nodular	241 (27.9)	198 (25.2)	39 (53.4)	4 (66.7)

EOR, extent of resection; GTR, gross total resection; STR, subtotal resection.

Cases broken down by MRI-defined and intraoperatively defined EOR. Percentages are calculated over the total number of cases in each column. One patient did not have an operative report available; MRI in this patient showed no residual.

### Primary Analysis

Table 3 outlays the impact of EOR on OS and IC-PFS in multivariable analysis. Presence of residual on MRI was associated with decreased IC-PFS (hazard ratio [HR], 1.32, 95% CI 1.13-1.55, *P* < .001) and OS (HR 1.28, CI 1.08-1.53, *P* = .005). The full multivariable model for the primary analysis is presented in **Supplemental Digital Content 1** (http://links.lww.com/NEU/E821).

**TABLE 3.** Multivariable Analyses of EOR vs Outcomes

	IC-PFS	OS
Intraoperatively assessed EOR (any residual)	1.24 (0.62-1.06), *P* = .12	1.50 (1.13-2.00), *P* = .006
MRI-assessed EOR (any residual)	1.32 (1.13-1.55), *P* < .001	1.28 (1.08-1.53), *P* = .005
Sensitivity analysis reclassifying dubious/minimal residual (n = 73) as gross total resection	1.32 (1.13-1.53) *P* < .001	1.26 (1.06-1.49), *P* = .01
Shape of residual on MRI (compared with no residual)		
Linear	1.33 (1.11-1.59), *P* = .002	1.21 (.99-1.48), *P* = .06
Nodular	1.30 (1.07-1.57), *P* = .007	1.33 (1.07-1.65), *P* = .01
Irregular	1.66 (1.03-2.70), *P* = .04	1.99 (1.22-3.25), *P* = .006

BM, brain metastasis; EOR, extent of resection; IC-PFS, intracranial progression-free survival; OS, overall survival.

In multivariable analysis, the effect size of EOR was corrected for age, performance status, number of BMs, size of the resected BM, infratentorial vs supratentorial location, primary tumor origin, extracranial metastases, presence of a targetable mutation, newly diagnosed vs recurrent BM status, year of surgery, and adjuvant radiation.

### Secondary Analyses

Sensitivity analysis reclassifying the 73 cases with dubious residuals as GTR yielded comparable results. Residual was associated with worse outcomes regardless of shape (linear, nodular, or irregular). The surgeon-described residual in the operative report predicted OS (HR 1.50, CI 1.13-2.00, *P* = .006), but not IC-PFS (*P* = .12; Table 3).

### Leptomeningeal Disease

For 821 patients, reliable follow-up on LMD development over the disease course was available. Of these, 111 (13.5%) developed LMD, which was more prevalent in the STR group (15.5%) than in the GTR group (10.7%). STR was associated with higher LMD occurrence in multivariable analysis (odds ratio 1.74, CI 1.10-2.76, *P* = .02; **Supplemental Digital Content 1** [http://links.lww.com/NEU/E821]).

### Subgroup Analysis

**FIGURE 1.**
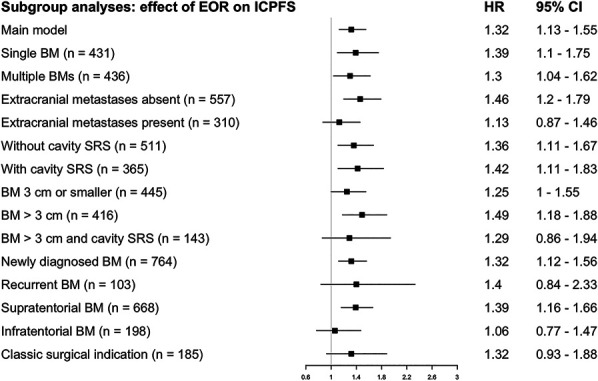
Stratified subgroup analyses (IC-PFS). The classical surgical indication denotes patients with a single, newly diagnosed BM that is >3 cm in diameter. BM, brain metastasis; EOR, extent of resection; IC-PFS, intracranial progression-free survival; SRS, stereotactic radiosurgery.

**FIGURE 2.**
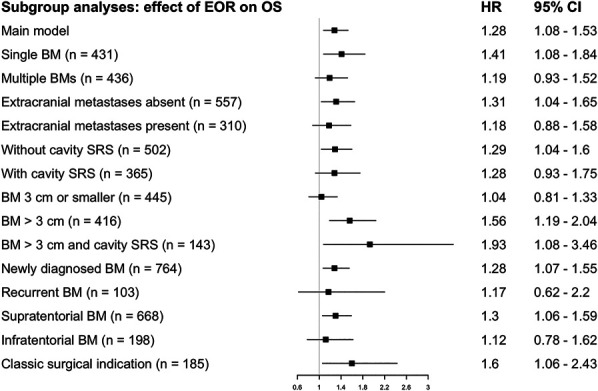
Stratified subgroup analyses (OS). The classical surgical indication denotes patients with a single, newly diagnosed BM that is >3 cm in diameter. BM, brain metastasis; EOR, extent of resection; OS, overall survival; SRS, stereotactic radiosurgery.

We performed a post hoc exploration of the impact of GTR vs STR on IC-PFS/OS in different subgroups. Detailed results are presented in Figures 1 and 2. In short, the alleged benefit of GTR was strongest in patients with brain-only disease (no extracranial metastases), supratentorial BMs, and BMs >3 cm. On the other hand, the impact of GTR was similar between patients with or without cavity SRS. Because some centers only offer resection + SRS for BMs >3 cm (and upfront SRS for smaller tumors), we explored the subgroup with BMs >3 cm receiving resection + SRS. In this subgroup, results were comparable with the primary analysis for IC-PFS (HR 1.29, CI 0.86-1.94), but the association between EOR and OS was stronger than in the main analysis (HR 1.93, CI 1.08-3.46; n = 143). Last, in the subgroup of patients with the “traditional” indication for resection, ie single, newly diagnosed BMs >3 cm, we observed similar results to the main analysis (IC-PFS HR 1.32, CI 0.93-1.88; OS HR 1.60, CI 1.06-2.43; n = 185).

### GTR vs STR + SRS

We were interested in investigating whether cavity SRS salvages the harm of residual tumor after resection. Divided into treatment groups, patients either underwent GTR with SRS (n = 161), GTR without SRS (n = 184), STR with SRS (n = 204), or STR without SRS (n = 316). Compared with GTR (with/without SRS), patients who underwent STR + SRS had worse IC-PFS (HR 1.35, *P* = .04) and a nonsignificant tendency toward worse OS (HR 1.29, *P* = .10). Moreover, receiving STR + SRS was associated with higher odds of LMD (odds ratio 1.92, CI 1.04-3.55, *P* = .04) compared with GTR. STR without SRS was also associated with worse IC-PFS (HR 1.34, *P* = .004) and OS (HR 1.28, *P* = .02) compared with GTR (Table 4).

**TABLE 4.** GTR vs STR With or Without SRS

Treatment	Median OS (months)	OS (HR, CI)	Intracranial recurrence, N (%)	Median time to intracranial recurrence (months)	IC-PFS HR
GTR (n = 345)	12.0 (5.4-22.5)	Reference	152 (44.1)	7.9 (3.9-14.4)	Reference
STR + SRS (n = 204)	14.0 (6.7-26.1)	1.29 (0.95-1.76), *P* = .10	122 (59.8)	6.0 (3.6-13.4)	1.35 (1.02-1.66), *P* = .04
STR, no SRS (n = 316)	7.1 (3.1-16.9)	1.28 (1.03-1.58), *P* = .02	120 (38.0)	4.7 (2.6-9.3)	1.34 (1.10-1.64), *P* = .004

GTR, gross total resection; HR, hazard ratio; IC-PFS, intracranial progression-free survival; OS, overall survival; SRS, stereotactic radiosurgery; STR, subtotal resection.

The GTR group encompasses both GTR + SRS and GTR without SRS. Survival times are presented as medians and IQR.

## DISCUSSION

This study assessed the impact of EOR on survival outcomes in 867 patients with BM. After adjusting for confounders, the presence of residual tumor on postoperative MRI correlated with worse OS, IC-PFS, and LMD. GTR compared with STR + SRS was associated with better IC-PFS, less LMD, and a nonsignificant tendency toward better OS.

Some previous studies have asked whether GTR on postoperative MRI improves BM outcomes. Kamp et al^6^ examined 130 metastasectomies; GTR was significantly associated with intracranial and local recurrence, although no multivariable analyses are reported. Winther et al^7^ found a positive relation between GTR and OS in a multivariable analysis of 373 single patients with BM (HR 0.66, *P* = .003). Only 17% received SRS; 40% received no radiation. Jünger et al^8^ found no correlation between GTR and OS or local recurrence in 197 patients with a single newly diagnosed BM. Notably, 68% of patients in this cohort had uncontrolled systemic disease.

Others have reported GTR vs STR in patients with BM as a secondary analysis in the context of a different primary research question.^9-19^ **Supplemental Digital Content 1** (http://links.lww.com/NEU/E821) provides an overview of studies that we found which were published after 1990 and include >100 patients. Chaichana et al^13^ explored prognostic factors in 421 patients with BM; GTR was not associated with OS. Other studies (typically 100-200 patients) vary in results; some find no relation between EOR and outcomes,^10,14,16^ others find a univariable association that does not hold in multivariable analysis,^9,11,12^ while others do find a benefit in multivariable analysis.^15,18,19^ Only 3 studies consistently define EOR using postoperative MRI^13,15,17^; one of which describes a significant effect in multivariable analysis.^15^ An important limitation is the lack^9-12,14,16,18,19^ or low proportion (2%-11%)^15,17^ of patients who received postoperative SRS, with only Chaichana et al^13^ reporting this in 33% of patients. In summary, previous studies have limitations and are conflicting, with the largest study^13^ actually reporting no significant correlation.

Our study population was relatively diverse. Although most previous studies have included only patients with a single BM,^7,8,17^ many of our patients (n = 436) had multiple BMs; 103 patients also had recurrent BMs. We believe this better reflects modern neurosurgical practice where indications for resection are broader than single, newly diagnosed BMs. BMs in our population were relatively small; the lack of evidence for resection + SRS vs upfront SRS only for BMs <3 cm has resulted in practice variation for this group. Particularly in Europe, it is common to offer upfront SRS for BMs <3 cm. To ensure generalizable outcomes, we performed subgroup analyses accounting for this heterogeneity.

Secondary analyses yielded some interesting findings. GTR of the resected lesion did not make a bigger difference in outcomes in patients with one vs multiple BMs. On the other hand, GTR had a stronger association with outcomes in patients with brain-only disease vs those with extracranial metastases. This aligns with the higher percentage (60%-70%) of patients with extracranial disease in large studies that found nonsignificant results.^8,13^ GTR seemed to matter more for supratentorial than for infratentorial BMs. We are unaware of previous studies investigating this. Local/intracranial recurrence was similar for supra- vs infratentorial BMs in our series (data not shown). It should be noted that subgroup analyses were exploratory, and future studies could shed more light on this question.

Postoperative SRS had little effect on the association between GTR and IC-PFS/OS, and patients receiving GTR tended toward better outcomes than those receiving STR + SRS. The adoption of cavity SRS is arguably the most important development in BM surgery of the past decades and has become the standard of care since randomized studies demonstrated that postoperative SRS is superior to WBRT^20^ or no radiation.^21^ Previous studies that demonstrated a benefit to GTR had relatively small SRS groups (eg, n = 58/373 in Winther's study^7^ and n = 8/116 in Kamp's study^6^), leaving the benefit of EOR in the setting of cavity SRS an open question. Studies drawing on historical data often include patients treated before SRS became widely adopted. In our population, WBRT was gradually replaced by SRS over the study period. We included 365 patients with cavity SRS, which, to our knowledge, is the largest series addressing GTR vs STR. Our results suggest that postoperative SRS may not entirely salvage the harm of residual tumor after surgery. Mechanisms for this harm largely align with the drawbacks of postoperative SRS:^22^ (1) tumor cells (from a residual) may spill into the cerebrospinal fluid during/after surgery; (2) complications may delay time to SRS, giving residual more time to grow/spread; and (3) cavity size and shape are irregular and may continuously change after surgery and the planning computed tomography, potentially causing inadequate target coverage with SRS, increasing the risk of recurrence. For these reasons, preoperative SRS is increasingly proposed, and this technique has indeed been associated with lower local and leptomeningeal recurrence.^23^ The higher incidence of LMD after STR in our study supports this hypothesis and offers a potential explanation for the diminished survival in this group.

In our population, 39.9% of patients had no residual contrast-enhancing lesion. In literature, GTR percentages vary widely, from 20%^24^ to 100%.^19,25^ While GTR may traditionally be perceived as very common in BM surgery, and many studies report rates upward of 70% to 90%, the investigations that have looked at EOR in BM as their primary question actually report lower rates of around 60%.^6-8^ Perhaps these studies investigated GTR/STR cutoffs with more scrutiny, which may lead to categorizing some patients with very small residuals as STR even although they were regarded as GTR at first sight. We also encountered this in our series. We adopted a very conservative approach, classifying dubious cases as STR; classifying these cases as GTR would have put the GTR rate in our population at 48.1%. The discrepancy between intraoperatively assessed residual (90.8% GTR) and residual on MRI confirms that the former method is not ideal for estimating EOR.

### Limitations

Some limitations should be considered. The retrospective study design warrants cautious interpretation. We did not perform volumetric analyses; these have proven valuable in glioblastoma. Since BMs are typically spherical, diameter measurements make reasonable proxies for tumor volume. There is inherent uncertainty in classifying very small or dubious residuals. We addressed this by taking a conservative approach, classifying these cases as STRs, and performing sensitivity analyses. Last, we could not take postoperative immunotherapy into account, because this was administered in a small minority of patients in our historical cohort. Nowadays, many melanoma/non-small cell lung cancer patients receive immunotherapy at some point, which may interfere with BM residuals. The strengths of this study lie in the size and diversity of our population; subgroup analyses address heterogeneity and increase the comparability of our results with different institutional practices. Last, we performed multivariable analysis adjusting for relevant confounders, including molecular mutations, which have, to our knowledge, not been adjusted for in previous studies.

Our results suggest that developing strategies to manage residuals in BM surgery is worthwhile. Intraoperative MRI or the development of BM-specific dyes may help identify residual tumor more accurately during surgery. The relation between different surgical techniques (en bloc vs piecemeal) and EOR should be further explored in future studies. Supramarginal resection has been proposed to improve outcomes, although more investigation is needed. Preoperative SRS is another promising avenue, which may lead to lower local recurrence and LMD.^22,23^

## CONCLUSION

Residual tumor on MRI after BM resection significantly correlates with worse OS and IC-PFS, as well as a higher prevalence of LMD. The impact of EOR seemed strongest in large, supratentorial metastases and in patients without extracranial metastases. Compared with GTR, STR + SRS was also associated with worse outcomes. Developing strategies to increase the rate of GTR in BMs may be a worthwhile future endeavor.

## Supplementary Material

## SUPPLEMENTAL DIGITAL CONTENT

**Supplemental Digital Content 1. Table 1.** (A) Differences between patients who did or did not receive postoperative SRS. (B) Reasons for deferring radiation. **Table 2.** Results of the multivariable analyses for overall and intracranial progression-free survival and LMD. **Table 3.** Studies reporting the relation between the EOR and survival outcomes in >100 BMs patients.

## COMMENT

The authors are to be commended for a large brain metastases study that attempts to account for the multiple involved variables including primary disease type, tumor size, radiation type, and tumor location. By including 867 patients over a 14-year period, the study provides a broad stroke view of how subtotal vs gross total resection may impact overall survival in brain metastases and supports an operative goal of gross total resection for brain metastases when feasible. The present study suggests that the gross total resection of brain metastases improves overall survival and that the subtotal resection of brain metastases increases the risk for subsequent leptomeningeal disease.

Recent significant advances in the treatment and overall survival of systemic solid tumor cancers, the utility of intracranial stereotactic radiosurgery (SRS), and the heterogeneity of the data included make it difficult for the present results to be definitive. Patients as far back as 2004 were included and significant advances in systemic cancer treatment have occurred since that time. Further, the impact of specific genetic alterations on survival outcomes for particular cancer subtypes is established and not included in the study. Such information would not be available for tumors studied more than a decade ago. Intracranial stereotactic radiosurgery has revolutionized the treatment of brain metastases and is the standard treatment for operative cavities with and without residual disease (or prior to resection). That only 41% of the included patients received SRS raises a question of how applicable the presented data is to present day treatment strategies. Included were 445 surgical patients with brain metastases less than 3 cm, presumably including many patients that in the present day would undergo SRS alone without operation.

While the heterogeneity of the data can make it difficult to draw conclusions, the authors performed an exceptional effort through numerous multivariate analyses to attempt to account for the challenges of studying metastatic disease patients. This large study provides insight into outcomes for the operative management of brain metastases.

**Andrew Fabiano**

*Buffalo, New York, USA*

## Notes

Surgical resection of symptomatic brain metastasis (BM) with mass effect is a mainstay treatment for affected patients.

Even with advances in neurosurgical techniques, complete tumor removal is not always achieved.

The authors present a retrospective study of the association between the extent of resection (EOR) and outcomes after brain metastasis (BM) surgery in 867 patients.

Residual tumor after BM resection correlated with worse overall survival (OS), intracranial progression-free survival (IC-PFS), and leptomeningeal disease (LMD), after adjusting for known prognostic factors in multivariate analysis for BMs.

Neurologic death and LMD might explain the mechanism through which a residual tumor leads to unfavorable outcomes. With increasing recognition of neurotoxicity associated with whole brain radiotherapy (WBRT), a preference for postresection cavity radiosurgery (post-SRS) has emerged over WBRT.

Radiosurgery protocols are continuously evolving, and there is a growing trend toward applying preoperative radiosurgery in case of large metastases that require surgery for brain decompression.

Delivering presurgery SRS may also allow full coverage of the metastases, with the potential to increase tumor control by decreasing intraoperative seeding of viable tumor cells beyond the treated cavity and decreasing the risk of LMD.

Additionally, pre-SRS reduces the volume and radiation dose in the surgical cavity (planning target volume), as the surgical corridor is not included. This treatment strategy allows for targeting of the intact metastasis, with more precise SRS without a margin of brain tissue, reducing the risk of radionecrosis compared to postoperative treatment.

The combination of surgery, radiosurgery and medical oncological treatment, including personalized therapies based on molecular profiles, offers synergy between focal and systemic therapies, leading to longer periods free of neurological disease and better control of the primary disease.

I believe that the study does help to strengthen the guides of management of patients with large metastases, feasible for surgical treatment.

**Jesus Ramiro del Valle Robles**

*Mexico City, Mexico*
