# Supplementary material for: Impact of Extent of Resection on Survival in Brain Metastasis: An Analysis of 867 Patients
Source: Neurosurgery. 2025 Jun 23;98(1):127–34. doi: 10.1227/neu.0000000000003544 (PMC12680270; doi:10.1227/neu.0000000000003544)
Supplement: Supplementary file 1 [file neu-98-127-s001.docx]

**Supplemental Data 1.** Three tables.

**Table 1A**. Differences between patients who received different types of radiation

|  | **SRS (n = 365)** | **WBRT (n = 343)** | **None (n = 159)** | **P-value** |
| --- | --- | --- | --- | --- |
| Male | 144 (39.5) | 144 (42.0) | 72 (45.3) | .449 |
| Age (median, IQR) | 62 (55 - 68) | 59 (52 - 67) | 60 (53 - 69) | .071 |
| BM size in cm (median, IQR) | 2.8 (2.1 - 3.6) | 3.2 (2.4 - 4.0) | 3.2 (2.4 - 3.9) | <.001* |
| Infratentorial location (%) | 68 (18.6) | 93 (27.2) | 37 (23.3) | .025 |
| Number of BMs (%) |  |  |  | <.001* |
| 1 | 228 (62.5) | 133 (38.8) | 70 (44.0) |  |
| 2 | 80 (21.9) | 66 (19.2) | 27 (17.0) |  |
| 3 | 35 (9.6) | 37 (10.8) | 15 (9.4) |  |
| >3 | 22 (6.0) | 107 (31.2) | 47 (29.6) |  |
| Extracranial metastases (%) | 140 (38.4) | 123 (35.9) | 47 (29.6) | .155 |
| Origin (%) |  |  |  | .053 |
| Breast | 56 (15.3) | 43 (12.5) | 24 (15.1) |  |
| Colorectal | 5 (1.4) | 17 (5.0) | 6 (3.8) |  |
| Gynaecological | 26 (7.1) | 20 (5.8) | 5 (3.1) |  |
| Lung | 151 (41.4) | 167 (48.7) | 70 (44.0) |  |
| Melanoma | 61 (16.7) | 33 (9.6) | 22 (13.8) |  |
| Miscellaneous | 50 (13.7) | 49 (14.3) | 23 (14.5) |  |
| Renal | 16 (4.4) | 14 (4.1) | 9 (5.7) |  |
| Karnofsky Performance Status | 90 (80 - 100) | 80 (60 - 100) | 80 (70 - 100) | <.001* |
| Recurrent BM (%) | 30 (8.2) | 21 (6.1) | 52 (32.7) | <.001* |
| Year of diagnosis (median, IQR) | 2014 (2013 - 2016) | 2011 (2008 - 2013) | 2013 (2009-2015) | <.001* |

*Statistically significant after Bonferroni adjustment for multiple comparisons.

**Table 1B.** Reasons for receiving no postoperative SRS or WBRT

| **Reason for not receiving postoperative radiation** | **N** |
| --- | --- |
| Complications (related to surgery or extracranial cancer), poor performance status, and/or poor postoperative survival leading to delay or omission of radiation | 67 |
| Recurrent/progressive BM with previous radiation | 33 |
| Radiation deferred in favor of systemic therapy (e.g., targeted therapy, immunotherapy, trial regimens) | 19 |
| Patient preference | 5 |
| Other | 9 |
| Unknown | 25 |

**Table 2**. Results of the multivariable analyses for overall and intracranial progression-free survival and leptomeningeal disease

|  | **Intracranial progression-free survival (HR, 95% CI, P-value)** | **Overall survival (HR, 95% CI, P-value)** | **Leptomeningeal disease (OR, 95% CI, P-value)** |
| --- | --- | --- | --- |
| Residual on postop MRI | 1.32 (1.13 - 1.55), P <.001 | 1.28 (1.08 - 1.53), P = .005 | 1.74 (1.10 - 2.76), P = .02 |
| Age | 1.00 (.99 - 1.00), P = .41 | 1.01 (1.00 - 1.02), P = .01 | .97 (.96 - .99), P = .01 |
| KPS | .99 (.98 - .99), P <.001 | .98 (.98 - .99), P <.001 | 1.01 (1.00 - 1.03), P = .12 |
| Year of surgery | .98 (.96 - 1.01), P = .23 | .97 (.95 - 1.00), P = .09 | 1.03 (.95 - 1.11), P = .46 |
| Infratentorial location | 1.19 (.99 - 1.42), P = .06 | 1.22 (1.00 - 1.50), P = .05 | 1.49 (.91 - 2.44), P = .11 |
| Number of BMs | 1.02 (.99 - 1.04), P = .17 | 1.02 (1.00 - 1.05), P = .11 | 1.01 (.93 - 1.09), P = .88 |
| Max BM diameter | .98 (.92 - 1.05), P = .58 | .98 (.91 - 1.05), P = .50 | 1.10 (.91 - 1.31), P = .32 |
| Extracranial metastases | 1.30 (1.08 - 1.55), P = .005 | 1.31 (1.07 - 1.60), P = .008 | .70 (.42 - 1.15), P = .16 |
| Tumor origin (reference: breast) |  |  |  |
| Colorectal | 1.06 (.67 - 1.7), P = .79 | .99 (.60 - 1.63), P = .96 | 1.45 (.42 - 5.07), P = .56 |
| Gynaecological | .90 (.62 - 1.3), P = .57 | .84 (.55 - 1.29), P = .43 | 1.26 (.46 - 3.49), P = .65 |
| Lung | .87 (.68 - 1.12), P = .28 | .84 (.63 - 1.12), P = .24 | .92 (.47 - 1.79), P = .80 |
| Melanoma | 1.03 (.77 - 1.39), P = .83 | 1.13 (.81 - 1.58), P = .46 | .65 (.28 - 1.47), P = .30 |
| Renal cell | .86 (.63 - 1.16), P = .31 | .95 (.68 - 1.33), P = .76 | .96 (.42 - 2.21), P = .93 |
| Other | .85 (.56 - 1.3), P = .46 | .67 (.41 - 1.08), P = .10 | .23 (.03 - 1.88), P = .17 |
| Targetable mutation | .64 (.50 - .81), P <.001 | .64 (.48 - .84), P = .001 | 1.87 (1.06 - 3.31), P = .03 |
| Recurrent BM | 1.23 (.95 - 1.58), P = .12 | .76 (.56 - 1.02), P = .06 | 1.60 (.82 - 3.12), P = .17 |
| Adjuvant radiation (reference: none) |  |  |  |
| SRS | .62 (.49 - .79), P <.001 | 032 (.25 - .42), P <.001 | 2.68 (1.30 - 5.55), P = .008 |
| WBRT | .64 (.51 - .81), P <.001 | .59 (.46 - .75), P <.001 | 1.50 (.71 - 3.15), P = .28 |

Legend. *Statistically significant (threshold P < .05). KPS and year of surgery are linear variables, and reported HRs are per year/KPS point. BM =brain metastasis, CI =confidence interval, HR =hazard ratio, KPS =Karnofsky performance status; MRI =magnetic resonance imaging, SRS =stereotactic radiosurgery.

**Table 3**. Studies reporting the relation between the extent of resection and survival outcomes in > 100 brain metastasis patients.

| **Article** | **N** | **GTR (N, %)** | **GTR definition** | **Adjuvant RT** | **Outcome: impact of GTR vs STR on survival** | **Topic of publication** | **Remarks** |
| --- | --- | --- | --- | --- | --- | --- | --- |
| Winther 2022 | 373 | 293 (64) | Postop MRI; doubtful cases classified as STR | 16% SRT; 39% WBRT, 19% partial brain RT; 4% SRT + WBRT, 31% no RT, 8% unknown | HR .66, P =.03 in multivariable analysis | EOR in BMs | - Multivariable model: age, sex, performance status, histology, extracranial disease, chemotherapy prior to surgery, BM location, EOR |
| Junger 2021 | 197 | 123 (62) | Postop MRI | 25% WBRT, 72% partial brain RT, 3% SRS | Not significant in univariable analysis; P =.76, HR NR. | EOR in BMs | - 68% of patients had uncontrolled systemic disease |
| She 2018 | 122 | 49 (40) | Undefined | 45% WBRT, 55% no RT | 12.6 v 8.7 months median survival, P < .001, but not adopted into multivariable model | Outcomes and prognostic factors in surgical NSCLC BM patients | - Multivariable model: BM first presentation of disease, KPS, RPA, postoperative chemotherapy |
| Sivasanker 2018 | 124 | 118 (95) | Postop MRI or contrast CT; ratio unclear | 52% WBRT, 48% no RT | 12.5 v 4.3 months median survival, P =.19 | Outcomes and prognostic factors in surgical BM patients | - Study conducted in India with relatively short OS of 8.5 months  - Small STR percentage |
| Liu 2017 | 125 | 77 (61) | Undefined | 34% received RT, unspecified which | 16.2 v 12.3 months (P =.03) median survival, but NS in multivariable model (no HR reported) | Prognostic factors in surgical BM patients | - Multivariable model: KPS, RPA, GPA, number of BMs, EOR, extracranial mets, treatment pattern |
| Enders 2016 | 114 | 76 (67) | Surgeon-determined; operative report; GTR only if all metastases were removed | 73% WBRT, 17% no RT | 13.2 v 6.6 months median survival, P =.04, but not adopted into multivariable model | Outcomes and prognostic factors in surgical NSCLC BM patients | - Multivariable model: unclear, only surgery for primary tumor reported  - 95 pts received WBRT, no SRS |
| Kamp 2015 | 130 | 80 (62) | Postop MRI | 77% WBRT, 15% no RT, 8% SRS/focal RT | Significant (P <.0001) for in-brain progression; but no hazard ratio reported | EOR in BMs | - No multivariable model reported |
| Chaichana 2014 | 421 | 298 (71) | Postop MRI, 100% resection | 67% received any RT, 51% WBRT, 33% SRS | NS (not further specified) | Prognostic factors in RPA II BM patients | - EOR Not adopted into multivariable model |
| Jin 2013 | 186 | 116 (62) | Undefined | No adjuvant radiation in GTR v STR comparison group | 15 v 14 months median survival, p-value NR | Descriptive series of BMs as first presentation of lung cancer | - Only patients without adjuvant radiation were compared |
| Lee 2013 | 157 | 119 (76) | Postop MRI and operative report | 69% WBRT, 11% SRS | HR .60, P =.03^2^ in multivariable analysis | Outcomes in surgical BM patients | - Only patients w/o extracranial metastases included  - Multivariable model (histology, control of primary, number of BMs, EOR) |
| Hashimoto 2011 | 130 | 124 (95) | Undefined | 51% WBRT, 49% local brain RT (25 x 2 Gy) | HR .62, P =.33 | Adjuvant SRS vs WBRT after BM resection | - Multivariable model (size, histology, RPA class, type of adjuvant RT)  - Small STR percentage |
| Tendulkar 2006 | 271 | 216 (80) | Postop MRI and intraoperative assessment | 84% WBRT, including 2% with SRS boost | 1.6 v 8.7 months median survivial, P =.07 | RPA validation in surgical BM patients | - EOR not adopted into multivariable model |
| Agboola 1998 | 125 | 86 (69) | Surgeon-defined; not further specified | 90% postop WBRT, 10% preop WBRT | 1.1 v 8.1 months median survival; multivariable HR NR; P =.02 | Prognostic factors in surgical BM patients | - Multivariable model (age, KPS, histology, systemic disease, radiation dose, EOR) |
| Smalley 1992 | 229 | 142 (62) | Undefined | 45% WBRT, the rest no radiation | HR .52^1^, P =.001 in multivariable analysis | Role of adjuvant WBRT and prognostic factors in resected BMs | - Multivariable model (EOR, adjuvant RT, age, sex, primary tumor site, systemic disease, BM size and location)  - Only includes patients with a single BM |

**Legend.** Color coding: red-EOR reported as not significant; yellow-significant or trend in univariable analysis, but no significance in multivariable model reported; green-reported as significant in multivariable analysis.

^1^Derived from a reported beta-coefficient of -.66.

^2^Derived from a HR of 1.675 for STR vs GTR.

BM =brain metastasis, CT =computed tomography, EOR =extent of resection, GPA =graded prognostic assessment, GTR =gross total resection, HR =hazard ratio, KPS =Karnofsky performance status; MRI =magnetic resonance imaging, NR =not reported, NS =not significant, NSCLC =non-small cell lung cancer, RPA =recursive partitioning analysis, RT =radiotherapy, SRS =stereotactic radiosurgery.
